# Supplementary material for: Convergent genome evolution shaped the emergence of terrestrial animals
Source: Nature. 2025 Nov 12;649(8097):638–46. doi: 10.1038/s41586-025-09722-4 (PMC12804077; doi:10.1038/s41586-025-09722-4)
Supplement: Supplementary file 1 — Supplementary text, Supplementary figures, legends for supplementary tables and supplementary references. [file 41586_2025_9722_MOESM1_ESM.pdf]

nature portfolio

https://doi.org/10.1038/s41586-025-09722-4

Supplementary information

Convergent genome evolution shaped the emergence of terrestrial animals

In the format provided by the authors and unedited

Supplementary Information for

Convergent genome evolution shaped the emergence of terrestrial animals

Authors: Jialin Wei[1], Davide Pisani[1,2], Philip Donoghue[2], Marta Álvarez-Presas[3,4]*, Jordi Paps[1]*†

Affiliations:

[1]School of Biological Sciences, University of Bristol; Bristol, United Kingdom.

[2]School of Earth Sciences, University of Bristol; Bristol, United Kingdom.

[3]Institut de Biologia Evolutiva (CSIC-Universitat Pompeu Fabra), Barcelona, Spain.

[4]Current address: Departament de Biologia Evolutiva, Ecologia i Ciències Ambientals, Facultat de Biologia, Barcelona, Spain.

*The authors contribute equally to the work

†Corresponding author

Table of Contents

1. Supplementary Text 3

1.1 Novel genes of terrestrial lineages are different from those in aquatic lineages 3

1.1.1 Novel gene rates found in terrestrial lineages are significantly higher than in aquatic nodes 3

1.1.2 Functions derived from novel genes in terrestrial lineages are significantly distinct from those in aquatic nodes 3

1.2 Key biological functions evolved convergently through gene gains in terrestrialisation events 4

1.2.1 Total number of HGs performing key functions increased in terrestrial nodes 4

1.2.2 Detailed Interpretation of representative genes hitting terrestrialisation-linked GOs in human and fruit fly 4

1.2.3 Enriched GO terms of expanded HGs 5

1.3 Semi-terrestrial animals share more adaptations than fully terrestrial lineages 5

1.3.1 Principal Component Analysis of the GOs associated with novel and ancestral genes 5

1.3.2 Share GO terms of novel genes in semi-terrestrial groups 6

1.3.3 Share expanded HGs in semi-terrestrial lineages 6

1.3.4 Share expanded HGs in fully terrestrial lineages 7

1.4 Distinct functions are found in each terrestrial node 7

1.4.1 Bdelloidea 7

1.4.2 Clitellates 7

1.4.3 Stylommatophora 7

1.4.4 Nematoda 8

1.4.5 Tardigrada 8

1.4.6 Onychophora 8

1.4.7 Arthropoda 8

1.4.8 Tetrapoda 9

1.5 Comparison between freshwater and terrestrial groups 9

2. Supplementary Figures 10

Supplementary Figure 1 11

Supplementary Figure 2 12

Supplementary Figure 3 13

Supplementary Figure 4 14

Supplementary Figure 5 15

Supplementary Figure 6 16

Supplementary Figure 7 18

Supplementary Figure 8 19

Supplementary Figure 9 20

3. Supplementary Tables Descriptions 21

4. References 25

1. Supplementary Text

1.1 Novel genes of terrestrial lineages are different from those in aquatic lineages

Our observed data, which include terrestrial nodes nested within aquatic ones, lacks sufficient statistical power for standard parametric tests (e.g., t-tests, ANOVA). Instead, we used phylogeny-wide permutation tests, which determine the statistical significance of observed data by comparing it against an empirical null distribution generated from numerous random permutations of the original data (e.g., evolutionary rates of novel genes in a node, GOs in a node) with their labels (aquatic or terrestrial) reshuffled in each permutation. This allows us to test specific hypotheses about evolutionary patterns by breaking the observed correlation between variables while preserving their individual distributions.

1.1.1 Novel gene rates found in terrestrial lineages are significantly higher than in aquatic nodes

In the first permutation test, we evaluated if the number of novel genes emerging per million year (Myr) in terrestrial nodes was significantly higher than in aquatic nodes. We collected the rate of emergence of novel genes for 11 terrestrial nodes and randomly selected 11 aquatic nodes (Actinopterygii, Ambulacraria, Bivalvia, Branchiopoda, Chondrichthyes, Cnidaria, Decapoda, Platyhelminthes, Priapulida, Sabellida and Vetigastropoda). We calculated the observed total evolutionary rate in the 11 terrestrial nodes as the total number of novel HGs divided by total divergence time (rate=4.900). We then repeatedly drew 11 sets of aquatic nodes randomly (with replacement) from the aquatic pool to build 10,000 permutations, and recomputing the evolutionary rate (total novel HGs counts divided by total divergence time), producing a null distribution of novel gene rates in aquatic nodes. As shown in Extended Data Fig. 3a, the observed terrestrial rates (red bar) exceeds the aquatic permutation rates (one-tailed $p = 0.0015$). Thus, the observed higher novel gene rates found in terrestrial lineages are significantly higher than in aquatic nodes, even after correcting for divergence time. The expanded and contracted genes were inferred with CAFE5[1], whose birth–death model is intrinsically scaled by branch length and does not require any correction.

1.1.2 Functions derived from novel genes in terrestrial lineages are significantly distinct from those in aquatic nodes

The second permutation test assessed if the biological functions found in terrestrial nodes are significantly different from those in other nodes. We included lineages with the biggest taxon sampling from random aquatic lineages, including Actinopterygii, Ambulacraria, Bivalvia, Branchiopoda, Cnidaria, Decapoda and Platyhelminthes. First, we converted the Gene Ontology (GO) matrix derived from the novel genes for each lineages into a binary presence/absence matrix, then quantified the dissimilarity between terrestrial and aquatic GO term profiles by measuring the proportion of non-shared terms (Jaccard GO distance). Then we built 10,000 permutations reshuffling the aquatic/terrestrial labels between lineages and recalculating the Jaccard distance between the reshuffled aquatic and terrestrial lineages. This

generates a null distribution of GO distances between lineages whose habitat has been reshuffled (Extended Data Fig. 3b). The GO distances for the real observed data (0.583, red bar, empirical $p < 1 \times 10^{-4}$) are outside the permutation distribution, indicating that ~46% of GO terms are habitat-specific and that this degree of gene turnover is highly unlikely under random node choice. This demonstrates that GO terms derived from novel genes are functionally distinct between terrestrial and aquatic lineages.

1.2 Key biological functions evolved convergently through gene gains in terrestrialisation events

1.2.1 Total number of HGs performing key functions increased in terrestrial nodes

Our findings show that not only novel HGs associated with those 55 GOs ("most specific" GO functions shared in novel HGs, Fig. 3b) convergently emerge in different terrestrial nodes, but the total number of novel HGs performing these functions also increased during these transitions. For the 10 "most specific" GO terms derived from the 27 GO terms shared by all nodes (Fig. 3a), we compared their numbers in terrestrial nodes with the HGs in their ancestors performing the same functions (Extended Data Fig. 4). The nodes Bdelloidea, Clitellata, Tardigrada, Onychophora, *Armadillidium*, and Tetrapoda show higher HG numbers hitting these GO terms compared to their immediate aquatic ancestors. For example, the number of novel HGs hitting GO:0005886 plasma membrane in Tetrapoda (22 HGs) is twofold that of Osteichthyans (14 HGs) or Dipnotetrapodomorpha (15 HGs), and fourfold that of Sarcopterygii (4 HGs). In conclusion, this indicates that the number of HGs involved in these biological functions increase in several terrestrial animals. However, given the limited taxa samplings in Tardigrada (two genomes), Onychophora (one genome), and Armadillidium (one genome), comparisons of HG numbers in these groups should be interpreted with caution, as additional genomes of these lineages may further refine this pattern.

1.2.2 Detailed Interpretation of representative genes hitting terrestrialisation-linked GOs in human and fruit fly

Genes included in those 55 GO terms in humans (tetrapods) and the fruit fly (hexapods) highlight the importance of biological functions linked to terrestrialisation (Extended Data Table 1). Human genes such as *IL27*[2], *APOA2*[3,4] are involved in lipid metabolism. *APOA2*, in particular, plays a role in lipid transport and fat accumulation and has been positively selected in cetaceans during their secondary aquatic adaptation[4]. Terrestrial and freshwater animals typically have more short-chain polyunsaturated fatty acids (PUFAs), such as alpha-Linolenic acid, than long-chain PUFAs[5,6], suggesting that adaptations in lipid metabolism are critical for landing animals. Genes *OSM*, *XCL1*, *CXCL16*, *TNFSF18*, *FLT3LG*, *XCL2*, *CD1* family members, *PLAUR*, *LYPD3,* and *TMIGD2* play crucial roles in immunity and response to stimuli, key to the transition to a new environment[7]. The Gene *MPIG6B* regulates blood cell function critical for terrestrial adaptation, driving a decreasing in thrombocyte size in amphibians and reptiles, and further in fully terrestrial species, with a significant reduction in mammals where thrombocytes evolved into anucleated platelets. This not only expands the membrane surface area to target more viruses but also facilitates distribution in flowing blood[8]. In addition, *SPP1*

gene is involved in bone health[9], and the novel *ENAM* gene encodes a large protein in the enamel matrix for teeth development, which interestingly became inactivated in secondary aquatic species (Mysticeti) with the loss of enamel[10]. *GPR152*, a receptor located in the plasma membrane and expressed in the retina, functions in retinal cell-to-cell communication[11]. Reproductive strategies also greatly changed from water to land. Novel genes in this category include members of the *AKAP* family, *TBC1D21*, *DKKL1,* and *ZNF239*, with *ZNF239* being a transcription factor related to the onset of sexual maturation[12]. Additionally, the *HR* gene was the first gene identified to be related to hair-cycle regulation[13], which possibly had a role in the skin barrier in early tetrapods. Lastly, *PPP1R3F* is found in brain astrocytes and neurodevelopment, helping the brain to regulate the storage of glycogen for energy[14]. In fruit flies, examples of terrestrial novel genes in hexapods include reproductive genes such as *Pof*, vital for viability in both sexes and female oogenesis[15], and the transcription factor *MESR4*, which promotes differentiation in ovarian germline stem cells[16]. Notably, fruit fly ionotropic receptors (*Ir64a*, *Ir75d*, *Ir31a*, *Ir84a*, also called ligand-gated ion channels) and the *Gr39b* gene are amongst the terrestrial novelties. These genes are linked to the detection of olfactory stimuli[17], including promoting male courtship[18] and taste stimuli[19], respectively, revealing key roles that novel genes play in terrestrial adaptation.

1.2.3 Enriched GO terms of expanded HGs

We applied GO terms enrichment analysis for the expanded HGs, using the bilaterian ancestral genes as background. These analyses also support that stimulus response and ion transport are key functions related to terrestrialisation (Supplementary Figure 6, Supplementary Table 8). In this analysis, all nodes except Tardigrada, Arachnida and Myriapoda show enriched GO Terms. Remarkably, we found GO terms convergently shared in at least five terrestrial nodes. Among these GO terms, biological process related to response to stimuli (such as stress, chemical, nutrients, external and biotic stimulus) and monoatomic ion transport occupy an important position. Other shared enriched functions include plasma membrane and transmembrane transporter complex in cellular components and transmembrane transporter activity (such as ion channel activity) in molecular functions.

1.3 Semi-terrestrial animals share more adaptations than fully terrestrial lineages

1.3.1 Principal Component Analysis of the GOs associated with novel and ancestral genes

We first performed Principal Component Analysis (PCA) of the GOs associated with novel and ancestral genes (Supplementary Figure 8) of terrestrial animal clades, as an initial exploratory ordination. The principal components (PC1 and PC2) are axes that represent the directions of maximum variance in the data. In the PCA of GO terms derived from novel genes, bdelloid rotifers do not cluster with the other animals (Supplementary Figure 8c), possibly due to their high rate of horizontal gene transfer, large genome sizes, asexual reproduction and extreme stress tolerance[20]. After removing Bdelloidea (Supplementary Figure 8d), PC1 and PC2 were

dominated by nematodes and tetrapods, respectively, with the single onychophoran genome (indicated with a red dot) did not clustering with any group ($p<0.05$, Supplementary Table 12). Semi-terrestrial groups are significantly different from fully terrestrial lineages on PC1 ($p=1.278e-10$). The top 1% dominating differences in novel gene functions encompass features related to semi- and fully terrestrial habitats (Supplementary Table 13), such as response to osmotic stress, epithelium development, physical barriers, cuticle development, reproduction and visual development. Ancestral genes do not show any clear clustering, even after removing the highly sampled tetrapods (Supplementary Figure 8a and 8b); this was expected, as ancestral genes include all the protein-coding genes present in the LCA of a lineage, which may not have a strong terrestrialisation signal. Bdelloidea ($p<0.05$ on PC2, Supplementary Table 12) and nematodes ($p<0.05$ on PC1 and PC2, Supplementary Table 12) are isolated from others. Interestingly, tardigrades are clearly separate from all other lineages on PC2 ($p<0.05$, Supplementary Table 12), which might be explained by their ability to survive in environments where other animals cannot thus possessing unique molecular adaptations[21,22]. However, because shared absences might bias Euclidean-based PCA on binary presence/absence data, inflating similarity between groups that simply lack many of the same features, we further performed Principal Coordinates Analysis (PCoA) on Jaccard dissimilarities of functional terms in novel genes for the main analyses (Figure 5), which more robustly reflects the "shared presence" structure.

1.3.2 Share GO terms of novel genes in semi-terrestrial groups

For novel genes, we explored the gene gains within the two groups, semi/terrestrial and fully terrestrial. There are 16 GO terms shared by the novel genes in all five semi-terrestrial events, while these terms were not shared with fully terrestrial lineages (Supplementary Figure 9). Interestingly, our results show that circulatory system development is linked to novel genes in all semi-terrestrial species, including GO:2000181 negative regulation of blood vessel morphogenesis, GO:0016525 negative regulation of angiogenesis and GO:1901343 negative regulation of vasculature development. The circulatory system has co-evolved with respiratory systems, which are simpler in semi-terrestrial species. Clitellates, bdelloidea, nematodes and tardigrades rely on diffusion-based or integumentary respiration, while onychophorans possess a simple tracheal system. These adaptations are in contrast with the more complex vascular mechanisms seen in fully terrestrial species (e.g., pneumostome of land snails, tracheal system in arthropods and lungs in tetrapods). Novel genes that negatively regulate excessive blood vessel formation to maintain efficient gas exchange and nutrient transport in their semi-terrestrial habitats[23]. Additionally, due to high soil dependence, semi-terrestrial species might have adapted to cope with waterborne and soil-based pollutants (GO:0009636 response to toxic substance) and break down nutrients (GO:1901565 organonitrogen compound catabolic process, GO:0009057 macromolecule catabolic process and GO:0030163 protein catabolic process).

1.3.3 Share expanded HGs in semi-terrestrial lineages

We investigated convergence in expanded HGs of animals adapting to semi-terrestrial environments (Figure 4a). Our results indicate that the terrestrial pattern of semi-terrestrial

lineages evolves convergently, with shared expanded HGs involved in osmoregulation (Tyrosine signaling regulators[24] and TWiK family[25]), nutrient absorption (Cathepsin B-like cysteine proteinase for protein breakdown[26] and Sulfate permease for sulfate uptake[27]), muscle contraction (Myosin family[28]), energy conservation (Dehydrogenase[29]), detoxification (ABC transporter[30] located on membranes and Protein-disulfide reductase[31] for antioxidation) and response to stimuli (transient receptor potential (TRP) channel family responding to temperature, touch, pain, osmolarity and taste[32] and SET domain protein[33]).

1.3.4 Share expanded HGs in fully terrestrial lineages

Most shared adaptations among fully terrestrial lineages are found in arthropods (see 1.4.7). Only two expanded HGs are shared by *Armadillidium* and Stylommatophora: the sodium-dependent glucose transporter, involved in glucose transport[34], and TRP channels, ion channels functioning as stimulus sensors[35] (Figure 4a, Supplementary table 7). Therefore, fully terrestrial lineages do not display a global pattern.

1.4 Distinct functions are found in each terrestrial node

1.4.1 Bdelloidea

Bdelloid rotifers evolved exceptional stress tolerance mechanisms, including resistance to desiccation, extreme temperatures, and radiation, enabling them to thrive in harsh environments[20]. In our study, Bdelloid rotifers genomes[20] show enrichment in stress-response functions (20% of expanded HGs, Supplementary Table 15), indicating adaptations to hypoxia, osmotic stress, temperature, radiation, PH and more. Key genes include nhr-4879, Hr9680, CYP4V281, and MAP2K382, which play roles in stress response. Bdelloid novel core genes (Supplementary Table 17) include *nhr-48*[36], *Hr96*[37], *CYP4V2*[38] and *MAP2K3*[39], which play a critical role in stress response.

1.4.2 Clitellates

Clitellates adapted their nervous and muscular systems to cope with terrestrial challenges[40,41], enhancing locomotion and desiccation resistance. Clitellate terrestrial adaptations center on nervous system changes, with expanded HGs encoding proteins like Neuroglian and Agrin for neuromuscular junctions[42] (Supplementary Table 16). Their novel genes support neurotransmitter-mediated synaptic communication. Terrestrial traits like simplified ganglia and ventral nerve cords, which enable rapid responses to environmental change[40,41], while muscle-related expanded HGs like Tropomyosin and Calponin-homology (CH) domain-containing protein[43], encoded by two expanded HGs in clitellates, aid in locomotion for desiccation avoidance (e.g., burrowing).

1.4.3 Stylommatophora

Terrestrial snails' adaptations include shell and eggshell formation (Calcitonin receptor, Chitin-binding proteins) mucus secretion and aestivation[44]; expanded and novel core genes (Supplementary Table 16, Supplementary Table 17) encode proteins like Calcitonin receptor[45] and Chitin-binding type-2 domain-containing protein[46], which are involved in calcium

metabolism and shell mineralization or eggshells deposition in molluscs, similar to previous study[47]. In addition, mucus-related genes such as Glycosyltransferase and Beta-1,3-galactosyl-O-glycosyl-glycoprotein beta-1,6-N-acetylglucosaminyltransferase are inferred in expanded genes, indicating glycosylation, an essential step in mucin-type biosynthesis[48], as shown in earlier studies reporting mucin-related gene family expansions[49,50]. Moreover, estivation, an ideal survival strategy for dealing with arid conditions where snails secrete a mucus to cover the aperture of the shell and prevent water loss[51], is supported by the expansion of dehydrogenase gene family in our results. These genes help conserve energy, adjust glucose metabolism and enhance antioxidant defense[29], also supported by other studies[29,50].

1.4.4 Nematoda

Free-living nematodes adapt through cuticle-related genes for structural resilience, to cope with pollutants, and modulate moulting[52]. In agreement with previous study[53], we found cuticle-related functions (e.g., structural constituents of collagen, cuticulin-based cuticle, cuticle modulation[54]) emerged in the nematode LCA (Supplementary Tables 17 and 18). Expanded HGs like acetylcholine receptors facilitate neuromuscular functions for soil navigation and feeding[55,56] (Supplementary Table 16).

1.4.5 Tardigrada

Tardigrades are able to survive in extreme environments, particularly during anhydrobiosis[21]. Our findings revealed several new stress response genes in novel core genes (Supplementary Table 17), for instance, *YWHAQ* gene (osmotic stress and dehydration[57]), *CSNK* gene (oxidative stress response and ROS levels[58]), and *NPR* genes (salt-water balance and blood pressure[59]). Additionally, expanded HGs (Supplementary Table 16) in tardigrades include genes involved in Olfactory receptor, rhodopsin family, G-protein coupled receptor and opsin family (light-sensitive receptor[60]).

1.4.6 Onychophora

Onychophorans share key characteristics with arthropods such as exoskeleton, segmentation, respiration via tracheae and an open circulatory system. Interestingly, we found Onychophora shared ten expanded HGs with *Armadillidium*, some of which are responsible for adaptation to oxygen levels (Glutathione Peroxidase[61] and NADH-Ubiquinone Oxidoreductase[62]) and nutrient and ion uptake (Major Facilitator Superfamily[63]) (Supplementary Table 7).

1.4.7 Arthropoda

They are the most diverse animal phylum on Earth, originated in the sea and colonized the land several times independently. In this study, we focus on fully terrestrial clade: Hexapoda, Myriapoda and Arachnida first and *Armadillidium* later[64]. These lineages in arthropods exhibit convergent evolutionary traits for terrestrial adaptation, such as exoskeleton structure, water conservation and sensory development (Figure 4a, Supplementary Table 7). For instance, the wax layer of the exoskeleton is responsible for waterproofing, minimizing desiccation, and repairing cuticle abrasions[65], regulated by expanded gene O-acyltransferase WSD1 (shared in Myriapoda and Hexapoda), improving osmotic stress tolerance[66]. Also, Retinol-binding protein genes required in the retinal pigment cells expanded in Arthropoda terrestrial lineages, linking

to visual development to adapt to light conditions on land[67]. Other expanded gene families include those responsible for wing hinge (MADF domain[68]), endothelial cell proliferation (THAP domain[69]) and response to various stimulus (Regucalcin for oxidative stress and sperm quality[70], C2H2 domain for drought, excessive light and oxidative stress[71]), consistent with stress tolerance physiology in terrestrial arthropods[72]. Hexapods gave rise to the majority of terrestrial animal biodiversity[64]. Interestingly, we found that Hexapoda exhibit enriched GO annotations in expanded genes (Supplementary Table 15) related to moulting (e.g., terpenoid metabolic process, juvenile hormone metabolic process, sesquiterpenoid metabolic process and steroid metabolic process) and vison (e.g., rhodopsin biosynthetic process) in response to terrestrialisation. Moulting, triggered by steroid hormones[73] and regulated by terpenoid[74], allows hexapods to grow within the constraints of their exoskeletons[75], providing waterproof protection thus facilitating their survival in terrestrial environments. Additionally, the evolution of sophisticated visual system involving the light-sensing protein rhodopsin in visual phototransduction[76], enables hexapods to navigate and avoid predators on land.

1.4.8 Tetrapoda

Land vertebrates show both novel and expanded genes with enriched GO annotations related to immunity functions (Supplementary Table 18 for novel HGs, Supplementary Table 15 for expanded HGs), such as T cell co-stimulation, positive regulation of activated T cell proliferation, neutrophil degranulation and specific granule lumen. Interestingly, the last two annotations play essential roles in innate immunity[77]. In addition to novel genes (mentioned in chapter two, e.g., *OSM*, *XCL1*, *CXCL16* and *CD1*), expanded HGs also contribute to the immune system (Supplementary Table 16), involving the Ly-6/uPAR family[78], sialic-acid-binding immunoglobulin-like lectins[79] and mucins[80] and resistin[81]. Notably, most of these genes have been highlighted in innate immunity. Such adaptation was crucial as tetrapods adapted to terrestrial habitats, evolving a specialised and reinforced epidermis with active keratinization and a resistant outer stratum corneum[82], closely linked to innate immunity. To defend against pathogens spreading in terrestrial environment, innate immunity is essential to provide both physical and chemical barriers[83], as evidenced by our study.

1.5 Comparison between freshwater and terrestrial groups

We analysed the shared GO terms (Supplementary Figure 4) and shared PFAMs (Supplementary Table 5) of novel genes between terrestrial and freshwater species, mostly sister groups. We found freshwater functional terms overlapping with terrestrial ones, including GO terms such as response to stress, plasma membrane and protein-containing complex, and Pfams such as protein kinase and Trypsin (Supplementary Table 5), supporting the view that some major functional categories involved in terrestrialisation were present in freshwater ancestors and later refined for land adaptation.

2. Supplementary Figures

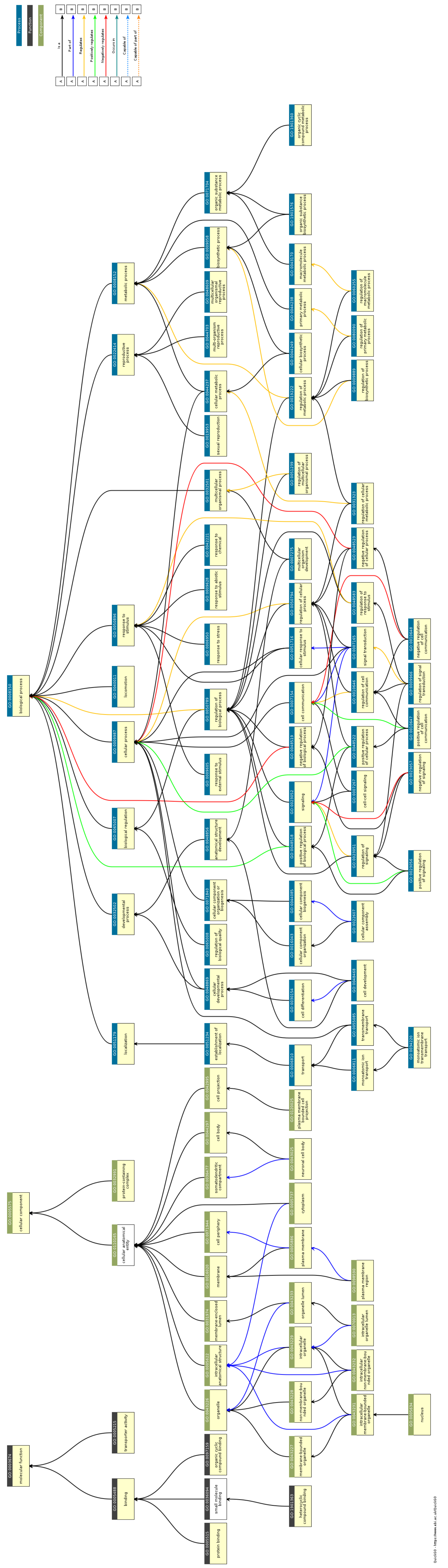

Supplementary Figure 1.

Relationship of 118 (27+91) Gene Ontology (GO) terms (yellow-filled boxes) associated with novel HGs shared by at least ten terrestrial nodes, including 27 GO terms shared across all nodes and 91 GO terms shared across all nodes except one. The figure created by GuickGO[84].

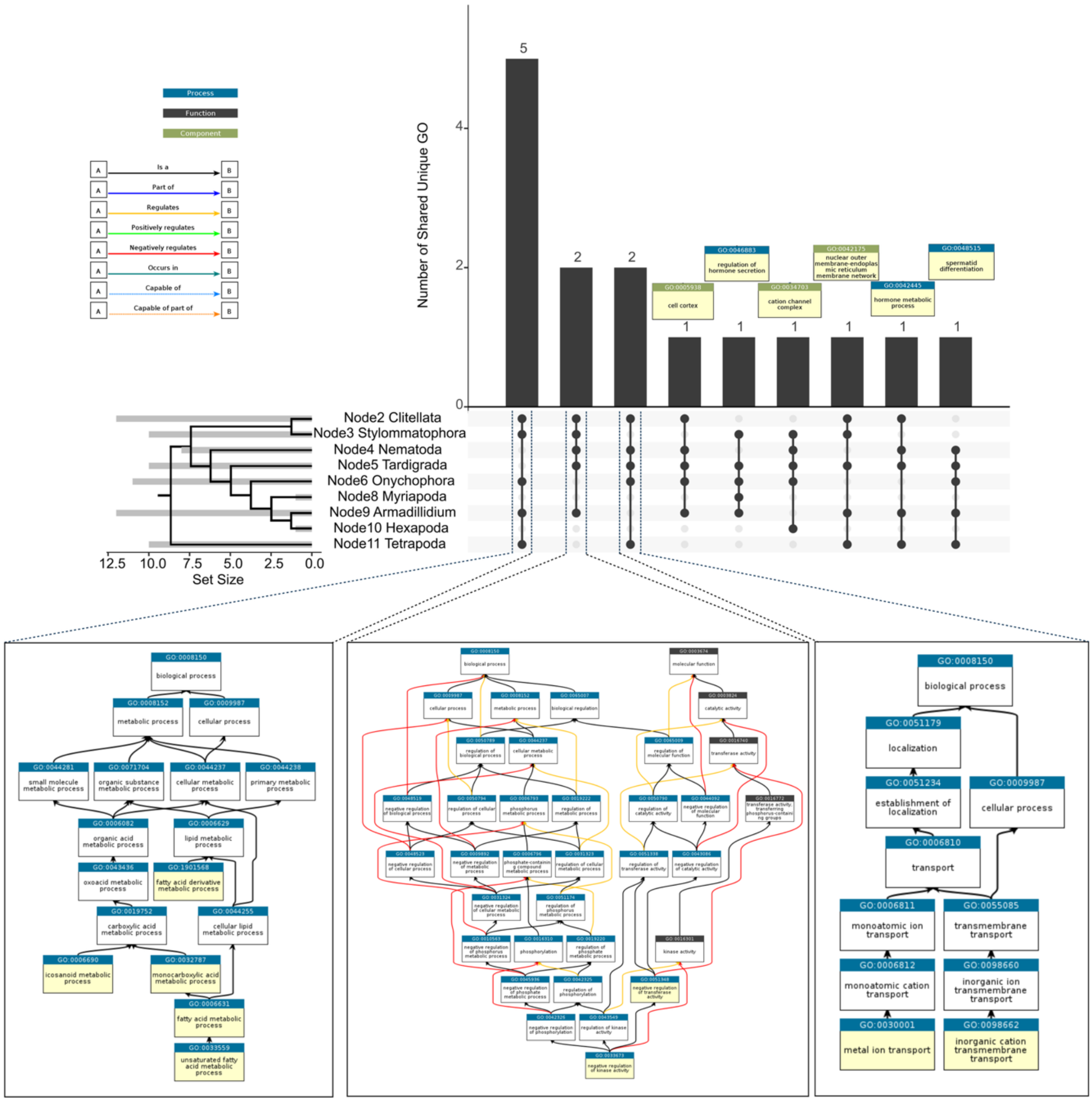

Supplementary Figure 2.

Analysis of "Unique GO" terms across terrestrial nodes. "Unique GO" terms were defined as GO terms associated with novel genes that are present in terrestrial nodes but absent in their ancestor nodes. The upset diagram shows the number of shared “Unique GO” across terrestrial nodes. All nodes except Node1 Bdelloidea and Node7 Arachnida contain "Unique GO". The three bottom panels display GO term relationship networks generated using QuickGO[84], with shared "Unique GO" terms highlighted in yellow-filled boxes. The UpSet diagram is generated by package UpSetR[85].

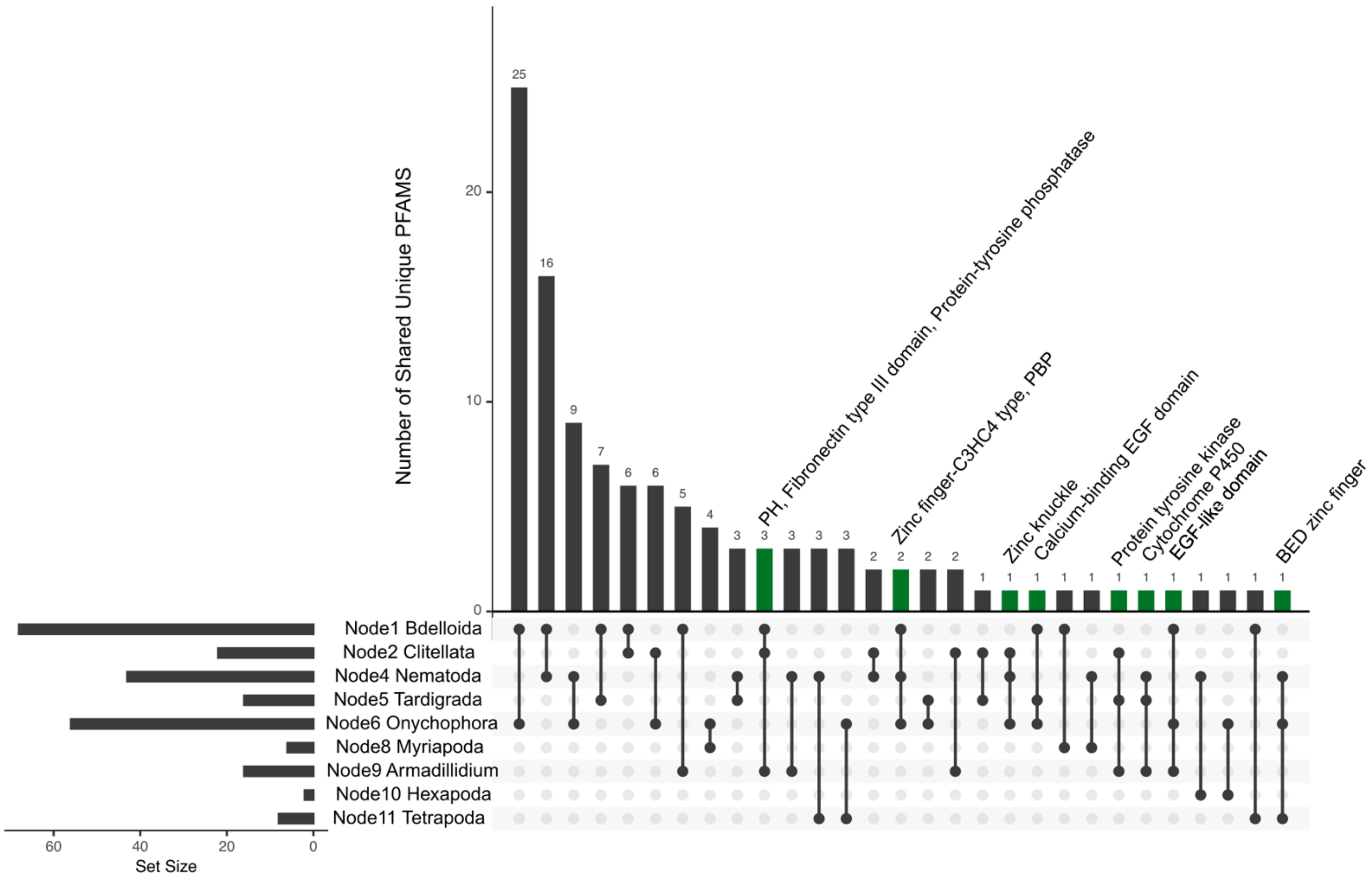

Supplementary Figure 3.

Analysis of "Unique Pfams" across terrestrial nodes. "Unique Pfams" were defined as Pfams associated with novel genes that are present in terrestrial nodes but absent in their ancestor nodes. The upset diagram shows the number of shared “Unique Pfams” across terrestrial nodes. All nodes except Node3 Stylommatophora and Node7 Arachnida contain "Unique GO". The shared "Unique Pfams" in at least three nodes are labelled with green bars.

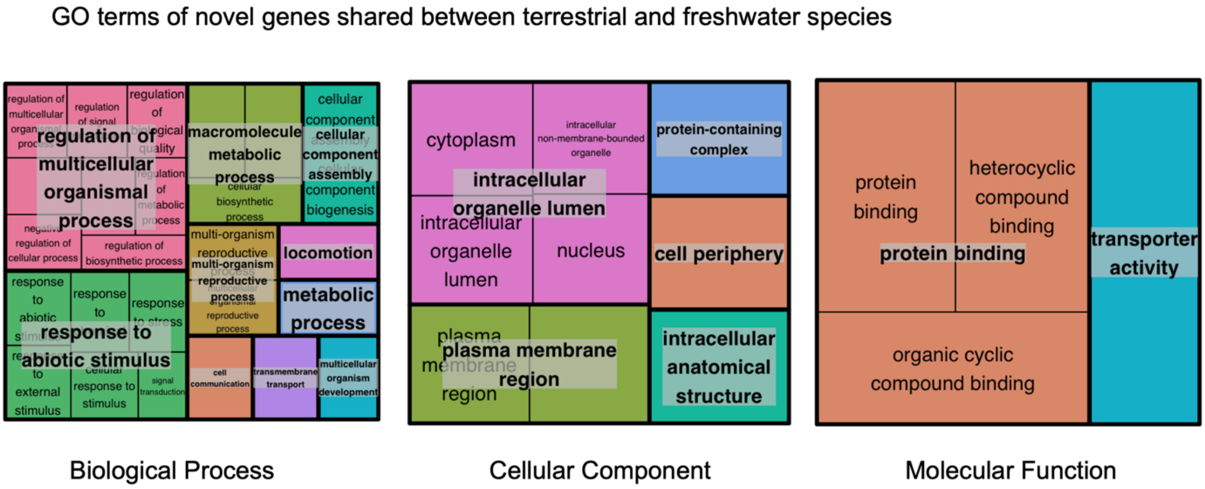

Supplementary Figure 4.

GO terms of novel genes shared between terrestrial and freshwater species

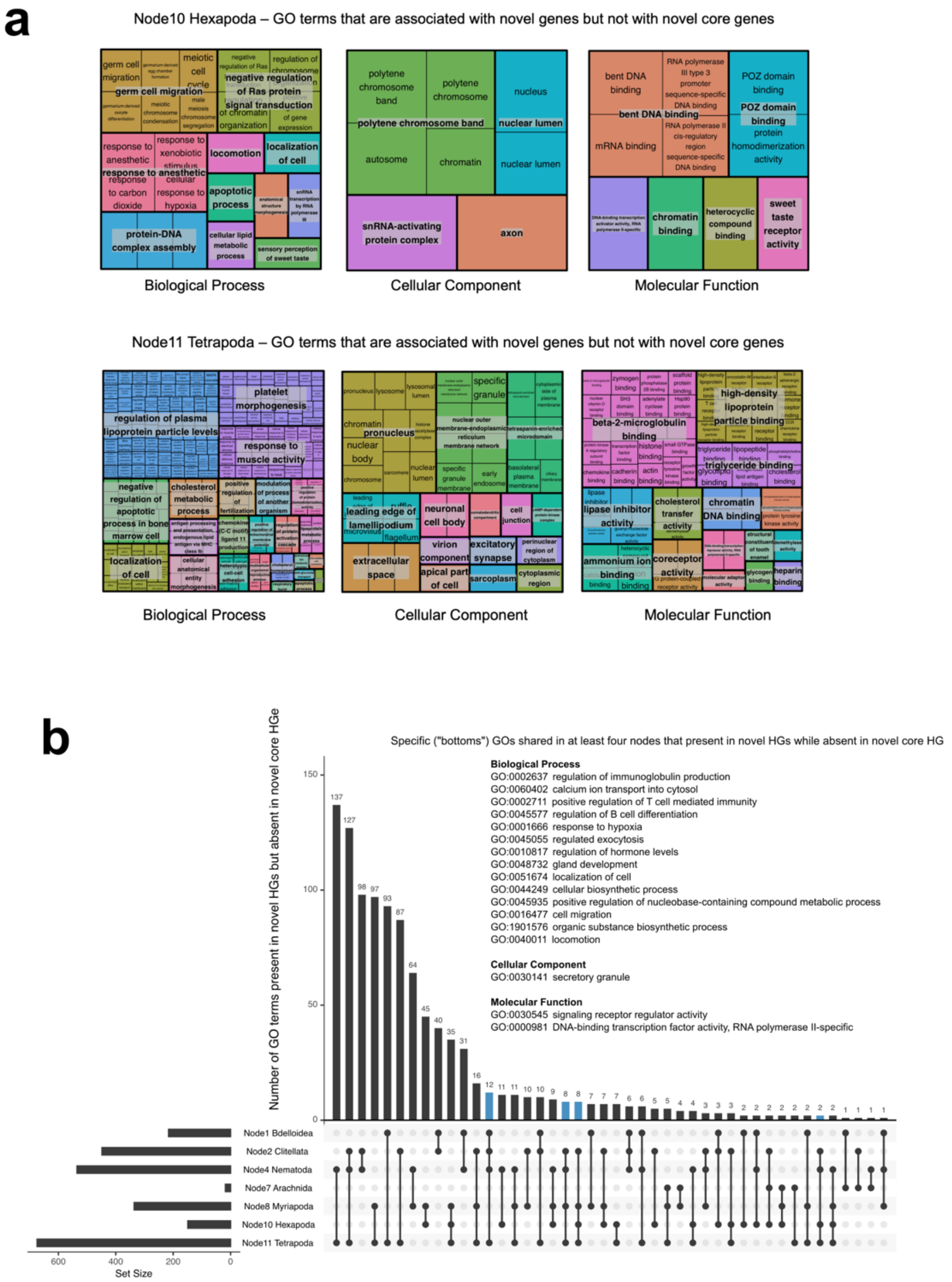

Supplementary Figure 5.

GO terms that are associated with novel genes but not with novel core genes, these functions were gained in the terrestrial ancestor but later lost. Our study distinguishes novel genes (gained in the last common ancestor a lineage, but that can be lost later) and novel core HGs (novel genes that are consistently retained across a lineage). Thus, the difference between novel and novel core highlights the differences between critical and non-critical functions. a) Examples of Hexapoda and Tetrapoda. b) Shared functions gained in the terrestrial ancestor but later lost

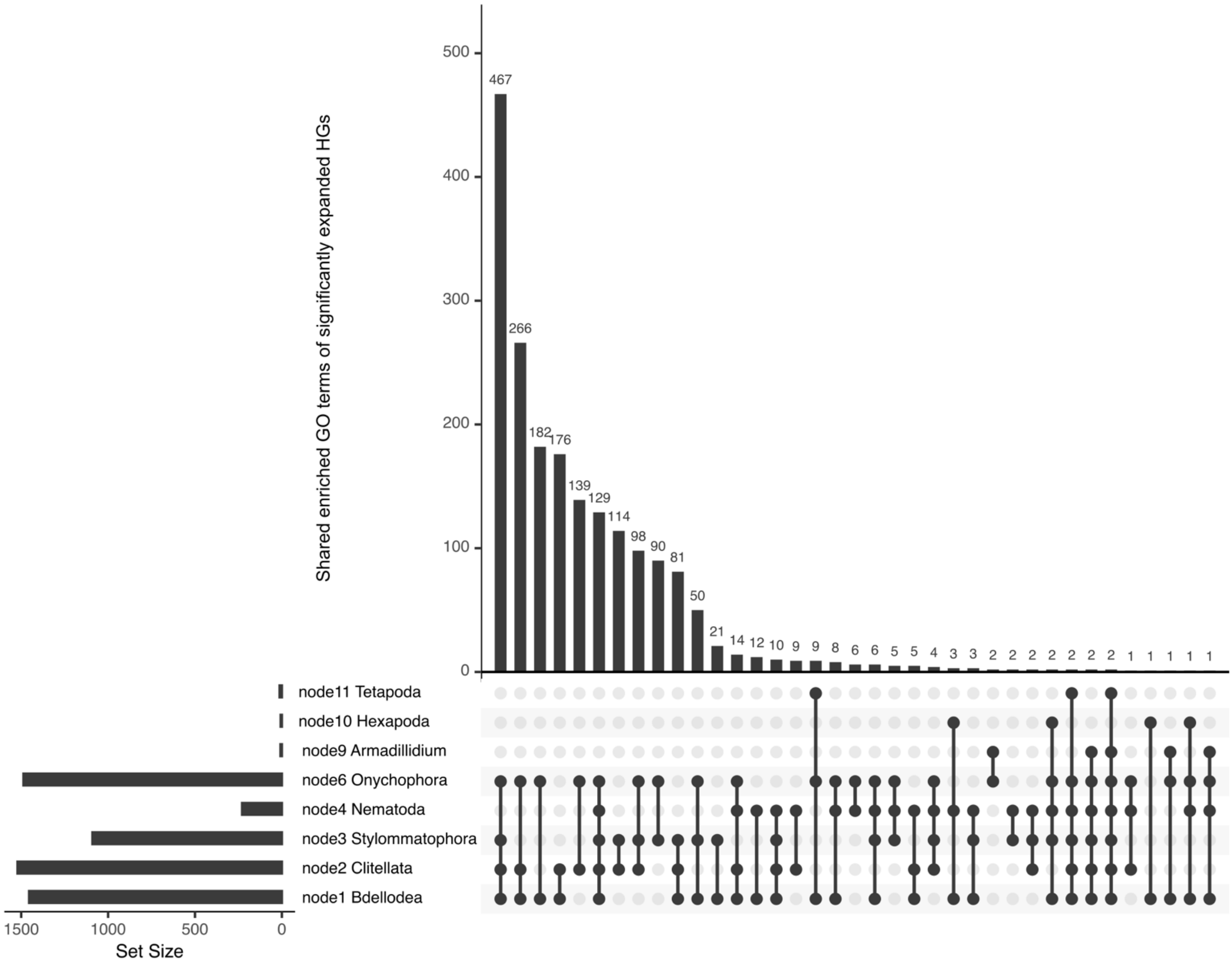

Supplementary Figure 6.

Shared enriched GO terms of significantly expanded HGs. All nodes except Tardigrada, Arachnida and Myriapoda show enriched GO Terms. The upset diagram displays the enriched GO terms of significantly expanded HGs shared among different combination of terrestrial nodes. We found GO terms convergently shared in at least five terrestrial nodes. Among these GO terms, biological process related to response to stimuli (such as stress, chemical, nutrients, external and biotic stimulus) and monoatomic ion transport occupy an important position. Other shared enriched functions include plasma membrane and transmembrane transporter complex in cellular components and transmembrane transporter activity (such as ion channel activity) in molecular functions. The UpSet diagram is generated by package UpSetR[85].

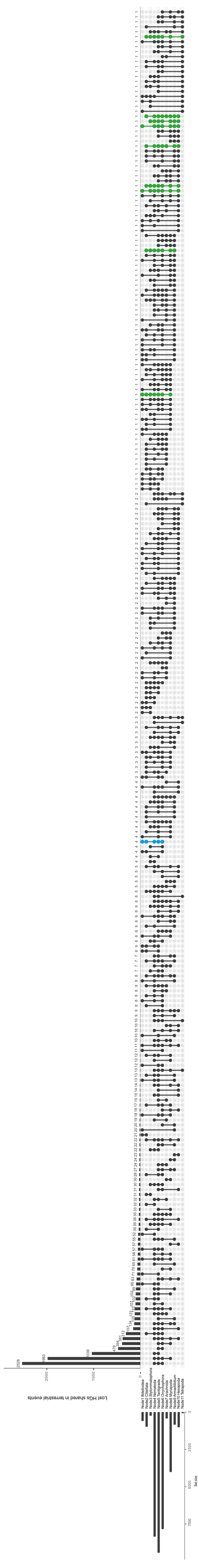
Lost HGs shared in terrestrial events
Set size
Node1 Bdelloidea
Node2 Clitellata
Node3 Stylommatophora
Node4 Nematoda
Node5 Tardigrada
Node6 Onychophora
Node7 Arachnida
Node8 Myriapoda
Node9 Armadillidium
Node10 Hexapoda
Node11 Tetrapoda

Supplementary Figure 7.

Shared Lost HGs across terrestrial nodes. The UpSet diagram displays the intersection of lost HGs across different combinations of terrestrial nodes. Lost HG shared in at least seven terrestrial nodes are highlighted by green dots, lost HG shared in semi-terrestrial nodes are highlighted by blue dots. The UpSet diagram is generated by package UpSetR[85].

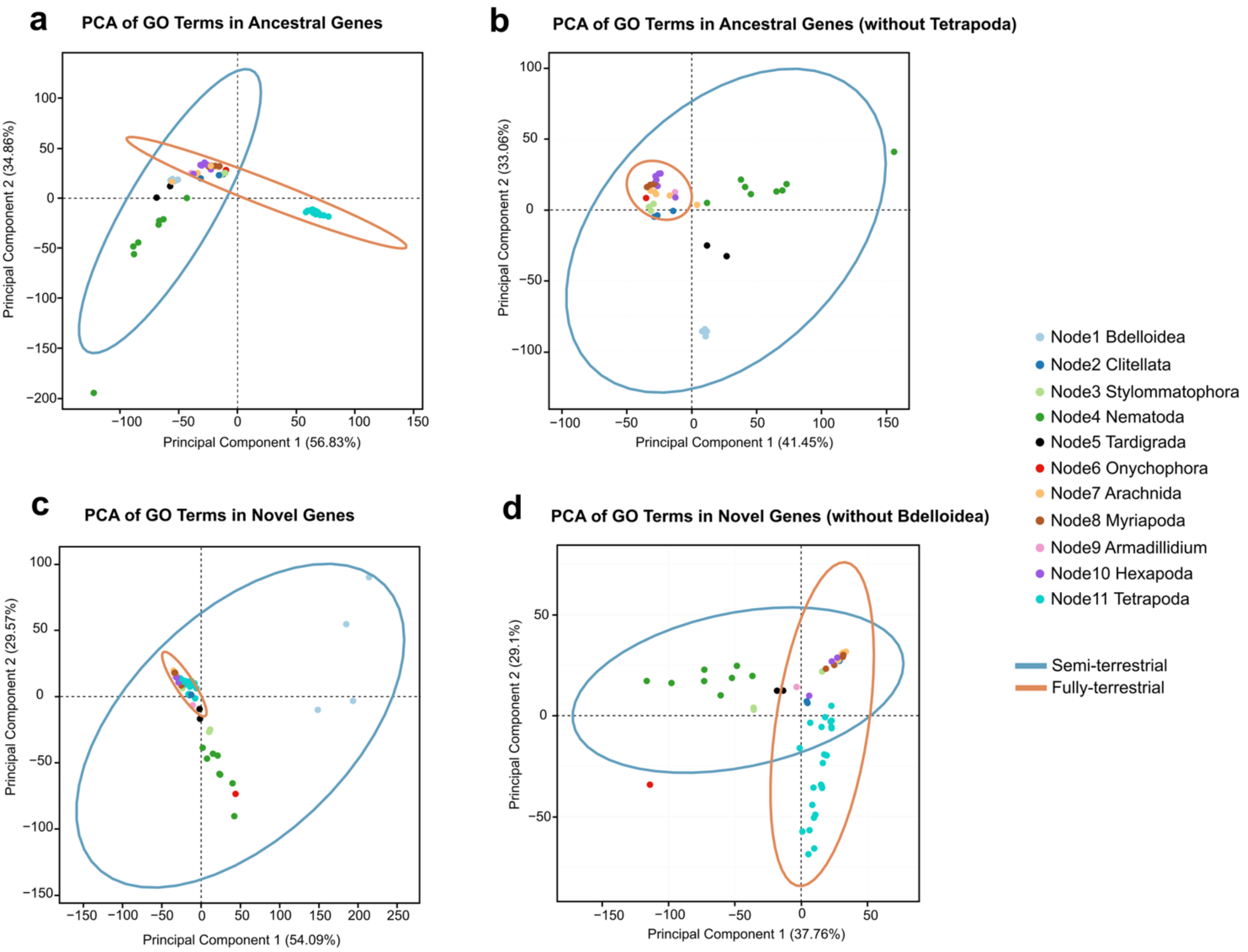

Supplementary Figure 8.

Principal Component Analysis (PCA) of GO terms hit ancestral and novel genes across terrestrialisation. Four PCA plots showing the distribution of GO terms across semi- and fully terrestrial groups, representing GO terms in **a,** Ancestral HGs, **b,** Ancestral HGs (removing Tetrapoda, **c,** Novel HGs, and **d,** Novel HGs (removing Bdelloidea). Each dot represents a species, coloured by taxonomic group as shown in the legend. Statistical ellipses are drawn to highlight semi-terrestrial group and fully terrestrial group. Ellipses were generated using normal distribution parameters to visualize the clustering patterns of taxonomic groups. The x and y axes represent the first two principal components (PC1 and PC2) with their respective explained variance percentages.

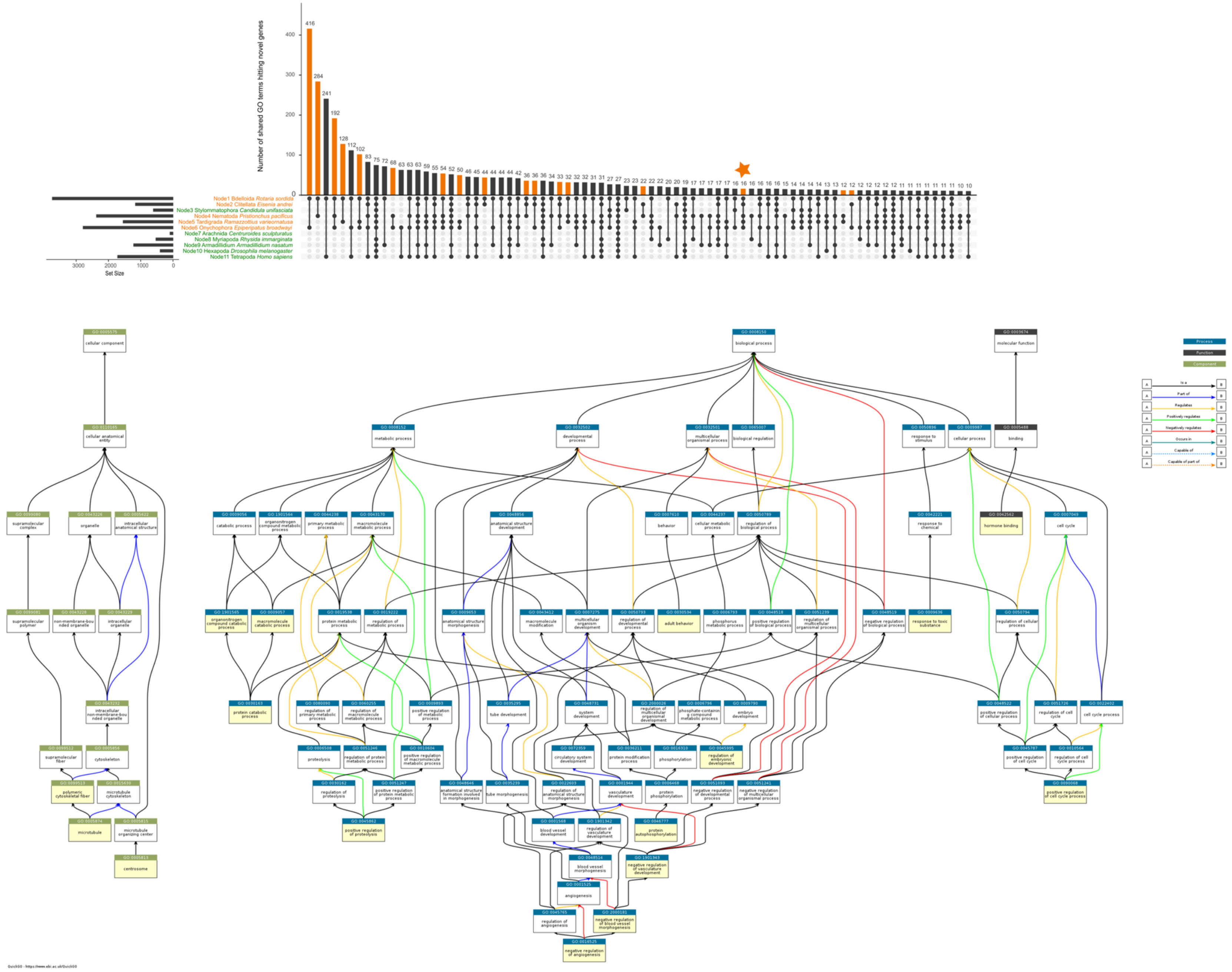

Supplementary Figure 9.

Shared GO terms associated with novel HGs among semi-terrestrial lineages and fully terrestrial lineages. The UpSet plot displays the distribution of shared GO terms, with orange bars highlighting GO terms shared among semi-terrestrial nodes. In the left panel, terrestrial nodes are color-differentiated to highlight semi-terrestrial nodes (orange text) and fully terrestrial nodes (green text). There are 16 GO terms shared in all semi-terrestrial lineages, marked by orange star. Their relationships and hierarchy shown below are created by QuickGO[84] in yellow-filled boxes. The UpSet diagram is generated by package UpSetR[85].

3. Supplementary Tables Descriptions

Supplementary Table 1.

154 genome samplings information, including their sources, BUSCO[86] results of genomes quality and habitats.

Supplementary Table 2.

Reconstruction of Homology groups (HGs) content, including eleven terrestrial nodes and their three immediate ancestors and aquatic sister groups. HG categories include novel, novel core, expanded, contracted, and lost HG.

Supplementary Table 3.

Evolutionary rates of novel HGs and novel core HGs. The numbers of novel and novel core HGs divided by branch length (in Myr) on our dated species tree.

Supplementary Table 4.

GO terms associated with novel or novel core HGs shared in at least ten terrestrial nodes, including the list of 27 GO terms (hit novel HGs) shared across all nodes and 91 GO terms (hit novel HGs) shared across all nodes except one, and list of 3 GO terms (hit novel core HGs) shared across all nodes and 23 GO terms (hit novel core HGs) shared across all nodes except one.

Supplementary Table 5.

Shared GO terms/Pfam domains associated with novel genes between terrestrial and freshwater species.

Supplementary Table 6.

GO terms with description that are associated with novel genes but not with novel core genes, these functions gained in the last common ancestor of each terrestrial clade but later lost.

Supplementary Table 7.

Uniprot annotation[87] of significantly (p<0.5) expanded HGs shared in terrestrial events. Including:

sheet1) Uniprot annotation of expanded HGs shared across four terrestrial events;

sheet2) Uniprot annotation of expanded HGs shared across semi-terrestrial lineages;

sheet3) Uniprot annotation of expanded HGs shared across fully terrestrial lineages;

sheet4) Uniprot annotation of expanded HGs shared between Onychophora and *Armadillidium*

Supplementary Table 8.

Enriched GO terms of significantly expanded HGs shared among at least five terrestrial events, with bottom (most specific) GO terms and their descriptions

Supplementary Table 9.

Uniprot annotation of Lost HGs shared among at least seven terrestrial events

Supplementary Table 10.

Uniprot annotation of significantly (*p*<0.5) contracted HGs shared in terrestrial events. Including:

sheet1) Uniprot annotation of contracted HGs shared across four terrestrial events;

sheet2) Uniprot annotation of contracted HGs shared across semi-terrestrial lineages;

sheet3) Uniprot annotation of contracted HGs shared across fully terrestrial lineages;

Supplementary Table 11.

Enriched functional terms separately in semi-terrestrial and fully terrestrial groups, including bottom (most specific) GO terms and their descriptions, and Pfam domains.

Supplementary Table 12.

Statistical summary (Tukey Test) of Principal Component Analysis (PCA) for GO terms hit novel HGs (removing Bdelloidea) and ancestral HGs(Removing Tetrapoda)

Supplementary Table 13.

Summary of GO terms with dominant contributions to Principal Component Analysis (PCA). The table presents GO terms with the highest loadings (top 1%) in both PC1 and PC2

Supplementary Table 14.

Uniprot annotation of lost HGs shared among all semi-terrestrial lineages, using *Capitella teleta* as example

Supplementary Table 15.

GO term enrichment analysis of significantly expanded HGs ($p<0.5$) in terrestrial nodes. The table are exported from REVIGO[88].

Supplementary Table 16.

Uniprot annotation/eggNOG-mapper[89] annotation of HGs showing significant expansion uniquely in specific terrestrial lineages. For instance, HGs that underwent expansion specifically in Clitellata but remained unexpanded in other terrestrial nodes.

Supplementary Table 17.

Uniprot annotation/eggNOG-mapper annotation of novel core HGs in each terrestrial node.

Supplementary Table 18.

GO term enrichment analysis of novel HGs in terrestrial nodes. Significant GO term enrichment was detected only in Nematoda and Tetrapoda, while other terrestrial nodes showed no significant GO term enrichment. The table are exported from REVIGO[88].

Supplementary Table 19.

Function similarity among three terrestrial windows, including:

Sheet1) Uniprot annotation of expanded HGs shared between Nematoda and Arthropoda;

Sheet2) GO terms hit novel HGs shared between Nematoda and Arthropoda;

Sheet3) Uniprot annotation of expanded HGs shared between Tetrapoda and Clitellata;

Sheet4) Uniprot annotation of expanded HGs shared between Bdelloidea and Stylommatophora

Supplementary Table 20.

60 runs of CAFE 5[1] to test convergence and select the best parameters.

Supplementary Table 21.

BLASTp[90] results of novel core HGs validation. The results show the hit numbers and percentage of hit above threshold (Evalue <1e-10, identity > 50%)

Supplementary Table 22.

Statistics summary and comparison of consistency of results across six MCMCtree[91] runs. The table is generated by Tracer[92].

4. References

1 Mendes, F. K., Vanderpool, D., Fulton, B. & Hahn, M. W. CAFE 5 models variation in evolutionary rates among gene families. *Bioinformatics* **36**, 5516-5518 (2021). https://doi.org/10.1093/bioinformatics/btaa1022

2 Yang, Y., Liu, H. & Liu, D. Preventing high-fat diet-induced obesity and related metabolic disorders by hydrodynamic transfer of Il-27 gene. *Int. J. Obes. (Lond)* **47**, 413-421 (2023). https://doi.org/10.1038/s41366-023-01293-6

3 Endo, Y., Kamei, K. I. & Inoue-Murayama, M. Genetic signatures of lipid metabolism evolution in Cetacea since the divergence from terrestrial ancestor. *J. Evol. Biol.* **31**, 1655-1665 (2018). https://doi.org/10.1111/jeb.13361

4 Sun, Y. B. et al. Genome-wide scans for candidate genes involved in the aquatic adaptation of dolphins. *Genome Biol. Evol.* **5**, 130-9 (2013). https://doi.org/10.1093/gbe/evs123

5 Twining, C. W. et al. The evolutionary ecology of fatty-acid variation: Implications for consumer adaptation and diversification. *Ecol. Lett.* **24**, 1709-1731 (2021). https://doi.org/10.1111/ele.13771

6 Kabeya, N. et al. Genes for de novo biosynthesis of omega-3 polyunsaturated fatty acids are widespread in animals. *Sci. Adv.* **4**, eaar6849 (2018). https://doi.org/10.1126/sciadv.aar6849

7 Miller, M. M. et al. Characterization of two avian MHC-like genes reveals an ancient origin of the CD1 family. *Proc. Natl. Acad. Sci. USA* **102**, 8674-9 (2005). https://doi.org/10.1073/pnas.0500105102

8 Menter, D. G. et al. Of vascular defense, hemostasis, cancer, and platelet biology: an evolutionary perspective. *Cancer Metastasis Rev.* **41**, 147-172 (2022). https://doi.org/10.1007/s10555-022-10019-5

9 Lin, E. Y., Xi, W., Aggarwal, N. & Shinohara, M. L. Osteopontin (OPN)/SPP1: from its biochemistry to biological functions in the innate immune system and the central nervous system (CNS). *Int. Immunol.* **35**, 171-180 (2023). https://doi.org/10.1093/intimm/dxac060

10 Randall, J. G., Gatesy, J. & Springer, M. S. Molecular evolutionary analyses of tooth genes support sequential loss of enamel and teeth in baleen whales (Mysticeti). *Mol. Phylogenet. Evol.* **171**, 107463 (2022). https://doi.org/10.1016/j.ympev.2022.107463

11 MacIver, M. A., Schmitz, L., Mugan, U., Murphey, T. D. & Mobley, C. D. Massive increase in visual range preceded the origin of terrestrial vertebrates. *Proc. Natl. Acad. Sci. USA* **114**, E2375-E2384 (2017). https://doi.org/10.1073/pnas.1615563114

12 Mohamed, A. R. et al. Leveraging transcriptome and epigenome landscapes to infer regulatory networks during the onset of sexual maturation. *BMC Genomics* **23**, 413 (2022). https://doi.org/10.1186/s12864-022-08514-8

13 Abbasi, A. A. Molecular evolution of HR, a gene that regulates the postnatal cycle of the hair follicle. *Sci. Rep.* **1**, 32 (2011). https://doi.org/10.1038/srep00032

14 Liu, Z. et al. Hemizygous variants in protein phosphatase 1 regulatory subunit 3F (PPP1R3F) are associated with a neurodevelopmental disorder characterized by developmental delay, intellectual disability and autistic features. *Hum. Mol. Genet.* **32**, 2981-2995 (2023). https://doi.org/10.1093/hmg/ddad124

15 Larsson, J., Svensson, M. J., Stenberg, P. & Makitalo, M. Painting of fourth in genus Drosophila suggests autosome-specific gene regulation. *Proc. Natl. Acad. Sci. USA* **101**, 9728-33 (2004). https://doi.org/10.1073/pnas.0400978101

16 Szarka-Kovacs, A. B., Takacs, Z., Bence, M., Erdelyi, M. & Jankovics, F. Drosophila MESR4 Gene Ensures Germline Stem Cell Differentiation by Promoting the Transcription of bag of marbles. *Cells* **11** (2022). https://doi.org/10.3390/cells11132056
17 Ai, M. et al. Ionotropic glutamate receptors IR64a and IR8a form a functional odorant receptor complex in vivo in Drosophila. *J. Neurosci.* **33**, 10741-9 (2013). https://doi.org/10.1523/JNEUROSCI.5419-12.2013
18 Grosjean, Y. et al. An olfactory receptor for food-derived odours promotes male courtship in Drosophila. *Nature* **478**, 236-40 (2011). https://doi.org/10.1038/nature10428
19 Sung, H. Y. et al. Heterogeneity in the Drosophila gustatory receptor complexes that detect aversive compounds. *Nat. Commun.* **8**, 1484 (2017). https://doi.org/10.1038/s41467-017-01639-5
20 Wilson, C. G., Pieszko, T., Nowell, R. W. & Barraclough, T. G. Recombination in bdelloid rotifer genomes: asexuality, transfer and stress. *Trends Genet.* **40**, 422-436 (2024). https://doi.org/10.1016/j.tig.2024.02.001
21 Hashimoto, T. et al. Extremotolerant tardigrade genome and improved radiotolerance of human cultured cells by tardigrade-unique protein. *Nat. Commun.* **7**, 12808 (2016). https://doi.org/10.1038/ncomms12808
22 Clark-Hachtel, C. M. et al. The tardigrade Hypsibius exemplaris dramatically upregulates DNA repair pathway genes in response to ionizing radiation. *Curr. Biol.* **34**, 1819-1830 e6 (2024). https://doi.org/10.1016/j.cub.2024.03.019
23 Monahan-Earley, R., Dvorak, A. M. & Aird, W. C. Evolutionary origins of the blood vascular system and endothelium. *J. Thromb. Haemost.* **11 Suppl 1**, 46-66 (2013). https://doi.org/10.1111/jth.12253
24 Deleuze, C., Duvoid, A., Moos, F. C. & Hussy, N. Tyrosine phosphorylation modulates the osmosensitivity of volume-dependent taurine efflux from glial cells in the rat supraoptic nucleus. *J. Physiol.* **523 Pt 2**, 291-9 (2000). https://doi.org/10.1111/j.1469-7793.2000.t01-2-00291.x
25 Mecawi, A. S., Varanda, W. A. & da Silva, M. P. Osmoregulation and the Hypothalamic Supraoptic Nucleus: From Genes to Functions. *Front. Physiol.* **13**, 887779 (2022). https://doi.org/10.3389/fphys.2022.887779
26 Shompole, S. & Jasmer, D. P. Cathepsin B-like cysteine proteases confer intestinal cysteine protease activity in Haemonchus contortus. *J. Biol. Chem.* **276**, 2928-34 (2001). https://doi.org/10.1074/jbc.M007321200
27 Pilsyk, S. & Paszewski, A. Sulfate permeasesphylogenetic diversity of sulfate transport. *Acta Biochim. Pol.* **56**, 375-84 (2009). https://doi.org/10.18388/ABP.2009_2470
28 Hartman, M. A. & Spudich, J. A. The myosin superfamily at a glance. *J. Cell. Sci.* **125**, 1627-32 (2012). https://doi.org/10.1242/jcs.094300
29 Storey, K. B. & Storey, J. M. Aestivation: signaling and hypometabolism. *J. Exp. Biol.* **215**, 1425-33 (2012). https://doi.org/10.1242/jeb.054403
30 Schwartz, M. S. et al. Detoxification of multiple heavy metals by a half-molecule ABC transporter, HMT-1, and coelomocytes of Caenorhabditis elegans. *PLoS ONE* **5**, e9564 (2010). https://doi.org/10.1371/journal.pone.0009564
31 Si, M. et al. Overexpression of Mycothiol Disulfide Reductase Enhances Corynebacterium glutamicum Robustness by Modulating Cellular Redox Homeostasis and Antioxidant Proteins under Oxidative Stress. *Sci. Rep.* **6**, 29491 (2016). https://doi.org/10.1038/srep29491
32 Vriens, J., Nilius, B. & Vennekens, R. Herbal compounds and toxins modulating TRP channels. *Curr. Neuropharmacol.* **6**, 79-96 (2008). https://doi.org/10.2174/157015908783769644

33 Caro, E. et al. The SET-domain protein SUVR5 mediates H3K9me2 deposition and silencing at stimulus response genes in a DNA methylation-independent manner. *PLoS Genet.* **8**, e1002995 (2012). https://doi.org/10.1371/journal.pgen.1002995
34 Harada, N. & Inagaki, N. Role of sodium-glucose transporters in glucose uptake of the intestine and kidney. *J. Diabetes Investig.* **3**, 352-3 (2012). https://doi.org/10.1111/j.2040-1124.2012.00227.x
35 Zhang, M. et al. TRP (transient receptor potential) ion channel family: structures, biological functions and therapeutic interventions for diseases. *Signal Transduct. Target. Ther.* **8**, 261 (2023). https://doi.org/10.1038/s41392-023-01464-x
36 Jones, L. M., Rayson, S. J., Flemming, A. J. & Urwin, P. E. Adaptive and specialised transcriptional responses to xenobiotic stress in Caenorhabditis elegans are regulated by nuclear hormone receptors. *PLoS ONE* **8**, e69956 (2013). https://doi.org/10.1371/journal.pone.0069956
37 Ji, M. et al. A nuclear receptor HR96-related gene underlies large trans-driven differences in detoxification gene expression in a generalist herbivore. *Nat. Commun.* **14**, 4990 (2023). https://doi.org/10.1038/s41467-023-40778-w
38 Li, W. et al. The role of cytochrome P450 3A2 and 4V2 in response to high-temperature stress in Tetranychus truncatus (Acari: Tetranychidae). *Exp. Appl. Acarol.* **91**, 263-277 (2023). https://doi.org/10.1007/s10493-023-00837-9
39 Kim, Y. J. et al. Genome-wide RNA interference screening reveals a COPI-MAP2K3 pathway required for YAP regulation. *Proc. Natl. Acad. Sci. USA* **117**, 19994-20003 (2020). https://doi.org/10.1073/pnas.1915387117
40 Purschke, G. Sense organs and central nervous system in an enigmatic terrestrial polychaete, Hrabeiella perighndulata (Annelida)–implications for annelid evolution. *Invertebr. Biol.* **119**, 329-341 (2005). https://doi.org/10.1111/j.1744-7410.2000.tb00019.x
41 Purschke, G. Terrestrial polychaetes - models for the evolution of the Clitellata (Annelida)? *Hydrobiologia* **406**, 87-99 (1999). https://doi.org/10.1023/a:1003780032497
42 Bezakova, G. & Ruegg, M. A. New insights into the roles of agrin. *Nat. Rev. Mol. Cell. Biol.* **4**, 295-308 (2003). https://doi.org/10.1038/nrm1074
43 Banuelos, S., Saraste, M. & Djinovic Carugo, K. Structural comparisons of calponin homology domains: implications for actin binding. *Structure* **6**, 1419-31 (1998). https://doi.org/10.1016/s0969-2126(98)00141-5
44 Schweizer, M., Triebskorn, R. & Kohler, H. R. Snails in the sun: Strategies of terrestrial gastropods to cope with hot and dry conditions. *Ecol Evol* **9**, 12940-12960 (2019). https://doi.org/10.1002/ece3.5607
45 Cardoso, J. C. R. et al. The calcitonin-like system is an ancient regulatory system of biomineralization. *Sci. Rep.* **10**, 7581 (2020). https://doi.org/10.1038/s41598-020-64118-w
46 Jin, C., Zhao, J., Pu, J., Liu, X. & Li, J. Hichin, a chitin binding protein is essential for the self-assembly of organic frameworks and calcium carbonate during shell formation. *Int. J. Biol. Macromol.* **135**, 745-751 (2019). https://doi.org/10.1016/j.ijbiomac.2019.05.205
47 Sun, J. et al. Signatures of Divergence, Invasiveness, and Terrestrialization Revealed by Four Apple Snail Genomes. *Mol. Biol. Evol.* **36**, 1507-1520 (2019). https://doi.org/10.1093/molbev/msz084
48 Schwientek, T. et al. Control of O-glycan branch formation. Molecular cloning and characterization of a novel thymus-associated core 2 beta1, 6-n-

acetylglucosaminyltransferase. *J. Biol. Chem.* **275**, 11106-13 (2000). https://doi.org/10.1074/jbc.275.15.11106
49 Aristide, L. & Fernandez, R. Genomic Insights into Mollusk Terrestrialization: Parallel and Convergent Gene Family Expansions as Key Facilitators in Out-of-the-Sea Transitions. *Genome Biol. Evol.* **15** (2023). https://doi.org/10.1093/gbe/evad176
50 Liu, C. et al. Giant African snail genomes provide insights into molluscan whole-genome duplication and aquatic-terrestrial transition. *Mol. Ecol. Resour.* **21**, 478-494 (2021). https://doi.org/10.1111/1755-0998.13261
51 Storey, K. B. Life in the slow lane: molecular mechanisms of estivation. *Comp. Biochem. Physiol. A Mol. Integr. Physiol.* **133**, 733-54 (2002). https://doi.org/10.1016/s1095-6433(02)00206-4
52 Semprucci, F., Grassi, E. & Balsamo, M. Simple Is the Best: An Alternative Method for the Analysis of Free-Living Nematode Assemblage Structure. *Water* **14**, 1114 (2022). https://doi.org/10.3390/w14071114
53 Politz, S. M. & Philipp, M. Caenorhabditis elegans as a model for parasitic nematodes: a focus on the cuticle. *Parasitol. Today* **8**, 6-12 (1992). https://doi.org/10.1016/0169-4758(92)90302-i
54 Turek, M. & Bringmann, H. Gene expression changes of Caenorhabditis elegans larvae during molting and sleep-like lethargus. *PLoS ONE* **9**, e113269 (2014). https://doi.org/10.1371/journal.pone.0113269
55 Boulin, T. et al. Eight genes are required for functional reconstitution of the Caenorhabditis elegans levamisole-sensitive acetylcholine receptor. *Proc. Natl. Acad. Sci. USA* **105**, 18590-5 (2008). https://doi.org/10.1073/pnas.0806933105
56 Schafer, W. Nematode nervous systems. *Curr. Biol.* **26**, R955-R959 (2016). https://doi.org/10.1016/j.cub.2016.07.044
57 Gouraud, S. S., Yao, S. T., Heesom, K. J., Paton, J. F. & Murphy, D. 14-3-3 proteins within the hypothalamic-neurohypophyseal system of the osmotically stressed rat: transcriptomic and proteomic studies. *J. Neuroendocrinol.* **19**, 913-22 (2007). https://doi.org/10.1111/j.1365-2826.2007.01604.x
58 Hu, Y., Xu, Z., Pan, Q. & Ma, L. Casein kinase 1 gamma regulates oxidative stress response via interacting with the NADPH dual oxidase complex. *PLoS Genet.* **19**, e1010740 (2023). https://doi.org/10.1371/journal.pgen.1010740
59 Song, W., Wang, H. & Wu, Q. Atrial natriuretic peptide in cardiovascular biology and disease (NPPA). *Gene* **569**, 1-6 (2015). https://doi.org/10.1016/j.gene.2015.06.029
60 Terakita, A. The opsins. *Genome Biol.* **6**, 213 (2005). https://doi.org/10.1186/gb-2005-6-3-213
61 Lubos, E., Loscalzo, J. & Handy, D. E. Glutathione peroxidase-1 in health and disease: from molecular mechanisms to therapeutic opportunities. *Antioxid. Redox Signal.* **15**, 1957-97 (2011). https://doi.org/10.1089/ars.2010.3586
62 Brandt, U. & Zickermann, V. *Encyclopedia of Biophysics: NADH-Ubiquinone Oxidoreductase (Complex I)*. Chapter 26 (Springer Berlin Heidelberg, 2013).
63 Quistgaard, E. M., Low, C., Guettou, F. & Nordlund, P. Understanding transport by the major facilitator superfamily (MFS): structures pave the way. *Nat. Rev. Mol. Cell. Biol.* **17**, 123-32 (2016). https://doi.org/10.1038/nrm.2015.25
64 Lozano-Fernandez, J. et al. A molecular palaeobiological exploration of arthropod terrestrialization. *Philos. Trans. R. Soc. Lond. B. Biol. Sci.* **371** (2016). https://doi.org/10.1098/rstb.2015.0133
65 Locke, M. Secretion of Wax through the Cuticle of Insects. *Nature* **184**, 1967-1967 (1959). https://doi.org/10.1038/1841967a0

66 Abdullah, H. M. et al. Increased Cuticle Waxes by Overexpression of WSD1 Improves Osmotic Stress Tolerance in Arabidopsis thaliana and Camelina sativa. *Int. J. Mol. Sci.* **22** (2021). https://doi.org/10.3390/ijms22105173
67 Wang, T. & Montell, C. Rhodopsin formation in Drosophila is dependent on the PINTA retinoid-binding protein. *J. Neurosci.* **25**, 5187-94 (2005). https://doi.org/10.1523/JNEUROSCI.0995-05.2005
68 Shukla, V., Habib, F., Kulkarni, A. & Ratnaparkhi, G. S. Gene duplication, lineage-specific expansion, and subfunctionalization in the MADF-BESS family patterns the Drosophila wing hinge. *Genetics* **196**, 481-96 (2014). https://doi.org/10.1534/genetics.113.160531
69 Cayrol, C. et al. The THAP-zinc finger protein THAP1 regulates endothelial cell proliferation through modulation of pRB/E2F cell-cycle target genes. *Blood* **109**, 584-94 (2007).
70 Silva, A. M. S. et al. Overexpression of regucalcin mitigates the ageing-related changes in oxidative stress and sperm quality. *Theriogenology* **157**, 472-482 (2020). https://doi.org/10.1016/j.theriogenology.2020.08.028
71 Wang, K., Ding, Y., Cai, C., Chen, Z. & Zhu, C. The role of C2H2 zinc finger proteins in plant responses to abiotic stresses. *Physiol. Plant.* **165**, 690-700 (2019). https://doi.org/10.1111/ppl.12728
72 Teets, N. M. & Denlinger, D. L. Surviving in a frozen desert: environmental stress physiology of terrestrial Antarctic arthropods. *J. Exp. Biol.* **217**, 84-93 (2014). https://doi.org/10.1242/jeb.089490
73 Cheong, S. P., Huang, J., Bendena, W. G., Tobe, S. S. & Hui, J. H. Evolution of Ecdysis and Metamorphosis in Arthropods: The Rise of Regulation of Juvenile Hormone. *Integr. Comp. Biol.* **55**, 878-90 (2015). https://doi.org/10.1093/icb/icv066
74 Sharma, E., Anand, G. & Kapoor, R. Terpenoids in plant and arbuscular mycorrhiza-reinforced defence against herbivorous insects. *Ann. Bot.* **119**, 791-801 (2017). https://doi.org/10.1093/aob/mcw263
75 Liu, X., Zhang, J. & Zhu, K. Y. *Chitin in Arthropods: Biosynthesis, Modification, and Metabolism: Targeting Chitin-containing Organisms*. (Springer Singapore, 2019).
76 Sakai, K. et al. Drosophila melanogaster rhodopsin Rh7 is a UV-to-visible light sensor with an extraordinarily broad absorption spectrum. *Sci. Rep.* **7**, 7349 (2017). https://doi.org/10.1038/s41598-017-07461-9
77 Kobayashi, S. D., Voyich, J. M., Burlak, C. & DeLeo, F. R. Neutrophils in the innate immune response. *Arch. Immunol. Ther. Exp. (Warsz)* **53**, 505-17 (2005).
78 Loughner, C. L. et al. Organization, evolution and functions of the human and mouse Ly6/uPAR family genes. *Hum. Genomics* **10**, 10 (2016). https://doi.org/10.1186/s40246-016-0074-2
79 Crocker, P. R., Paulson, J. C. & Varki, A. Siglecs and their roles in the immune system. *Nat. Rev. Immunol.* **7**, 255-66 (2007). https://doi.org/10.1038/nri2056
80 Pelaseyed, T. et al. The mucus and mucins of the goblet cells and enterocytes provide the first defense line of the gastrointestinal tract and interact with the immune system. *Immunol. Rev.* **260**, 8-20 (2014). https://doi.org/10.1111/imr.12182
81 Li, Y. et al. Resistin, a Novel Host Defense Peptide of Innate Immunity. *Front. Immunol.* **12**, 699807 (2021). https://doi.org/10.3389/fimmu.2021.699807
82 Lillywhite, H. B. Water relations of tetrapod integument. *J. Exp. Biol.* **209**, 202-26 (2006). https://doi.org/10.1242/jeb.02007
83 Riera Romo, M., Perez-Martinez, D. & Castillo Ferrer, C. Innate immunity in vertebrates: an overview. *Immunology* **148**, 125-39 (2016). https://doi.org/10.1111/imm.12597

84 Binns, D. et al. QuickGO: a web-based tool for Gene Ontology searching. *Bioinformatics* **25**, 3045-6 (2009). https://doi.org/10.1093/bioinformatics/btp536
85 Conway, J. R., Lex, A. & Gehlenborg, N. UpSetR: an R package for the visualization of intersecting sets and their properties. *Bioinformatics* **33**, 2938-2940 (2017). https://doi.org/10.1093/bioinformatics/btx364
86 Manni, M., Berkeley, M. R., Seppey, M., Simao, F. A. & Zdobnov, E. M. BUSCO Update: Novel and Streamlined Workflows along with Broader and Deeper Phylogenetic Coverage for Scoring of Eukaryotic, Prokaryotic, and Viral Genomes. *Mol. Biol. Evol.* **38**, 4647-4654 (2021). https://doi.org/10.1093/molbev/msab199
87 UniProt, C. UniProt: the Universal Protein Knowledgebase in 2023. *Nucleic Acids Res.* **51**, D523-D531 (2023). https://doi.org/10.1093/nar/gkac1052
88 Supek, F., Bošnjak, M., Škunca, N. & Šmuc, T. REVIGO summarizes and visualizes long lists of gene ontology terms. *PLoS ONE* **6**, e21800 (2011). https://doi.org/10.1371/journal.pone.0021800
89 Cantalapiedra, C. P., Hernandez-Plaza, A., Letunic, I., Bork, P. & Huerta-Cepas, J. eggNOG-mapper v2: Functional Annotation, Orthology Assignments, and Domain Prediction at the Metagenomic Scale. *Mol. Biol. Evol.* **38**, 5825-5829 (2021). https://doi.org/10.1093/molbev/msab293
90 Bealer, K. et al. BLAST+: architecture and applications. *BMC Bioinformatics* **10**, 421 (2009). <http://dx.doi.org/10.1186/1471-2105-10-421>.
91 Alvarez-Carretero, S. et al. A species-level timeline of mammal evolution integrating phylogenomic data. *Nature* **602**, 263-267 (2022). https://doi.org/10.1038/s41586-021-04341-1
92 Rambaut, A., Drummond, A. J., Xie, D., Baele, G. & Suchard, M. A. Posterior Summarization in Bayesian Phylogenetics Using Tracer 1.7. *Syst. Biol.* **67**, 901-904 (2018). https://doi.org/10.1093/sysbio/syy032
